# Supplementary material for: Two-dimensional inorganic molecular crystals
Source: Nat Commun. 2019 Oct 17;10:4728. doi: 10.1038/s41467-019-12569-9 (PMC6797790; doi:10.1038/s41467-019-12569-9)
Supplement: Supplementary file 1 — Supplementary Information [file 41467_2019_12569_MOESM1_ESM.pdf]

Two-dimensional inorganic molecular crystals

Han et al.

Supplementary information for:

Two-dimensional inorganic molecular crystals

Wei Han[1]†, Pu Huang[2]†, Liang Li[1], Fakun Wang[1], Peng Luo[1], Kailang Liu[1], Xing Zhou[1], Huiqiao Li[1,3], Xiuwen Zhang[2], Yi Cui[3]★ and Tianyou Zhai[1]★

[1]State Key Laboratory of Material Processing and Die & Mould Technology, School of Materials Science and Engineering, Huazhong University of Science and Technology (HUST), Wuhan 430074, China

[2]Shenzhen Key Laboratory of Flexible Memory Materials and Devices, College of Electronic Science and Technology, Shenzhen University, Nanhai Avenue 3688, Shenzhen, Guangdong 518060, China

[3]Department of Material Science and Engineering, Stanford University, Stanford, CA, USA

†These authors contributed equally to this work.

★E-mail: zhaity@hust.edu.cn; yicui@stanford.edu

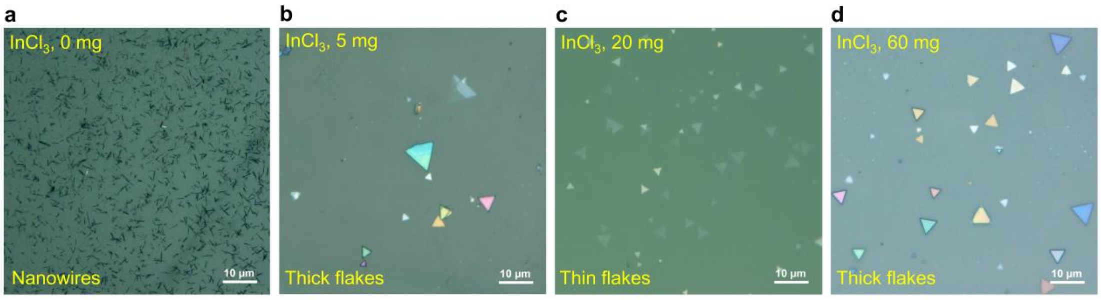

Supplementary Figure 1. Optical images of CVD grown Sb_2O_3 flakes using different amounts of passivators ($InCl_3$), showing the evolution of morphology, thickness, and nucleation density. (a-d) Typical optical images of the Sb_2O_3 flakes on mica using different amounts of $InCl_3$. (a) 0 mg, dense nanowires. (b) 5 mg, sparse thick flakes. (c) 20 mg, dense thin flakes. (d) 60 mg, dense thick flakes.

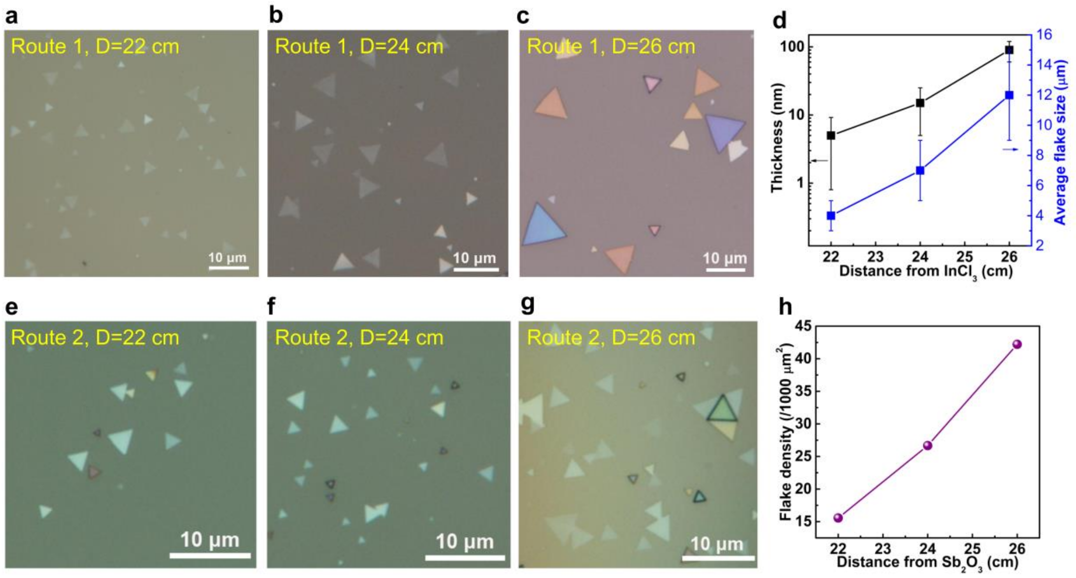

Supplementary Figure 2. Optical images of CVD grown Sb_2O_3 flakes deposited on different positions by two routes. (a-c) Typical optical images of triangular Sb_2O_3 flakes deposited on the mica of different positions (*D* = 22-26 cm, where *D* denotes the distance from $InCl_3$) in Route 1, showing a thickness and size evolution. (d) Plot of the thickness and average flake size of Sb_2O_3 flakes as a function of distance between substrates and $InCl_3$, revealing the minimum thickness down to ~0.71 nm and the maximum lateral size up to ~15 μm. Error bars are the range of thickness and size obtained from data of two measurements. (e-g) Optical images of Sb_2O_3 flakes deposited on different positions from Sb_2O_3 powder in Route 2, which show that the flake density increases with increasing the distance from Sb_2O_3 source. (h) Flake density of Sb_2O_3 plotted as a function of distances from Sb_2O_3 powder.

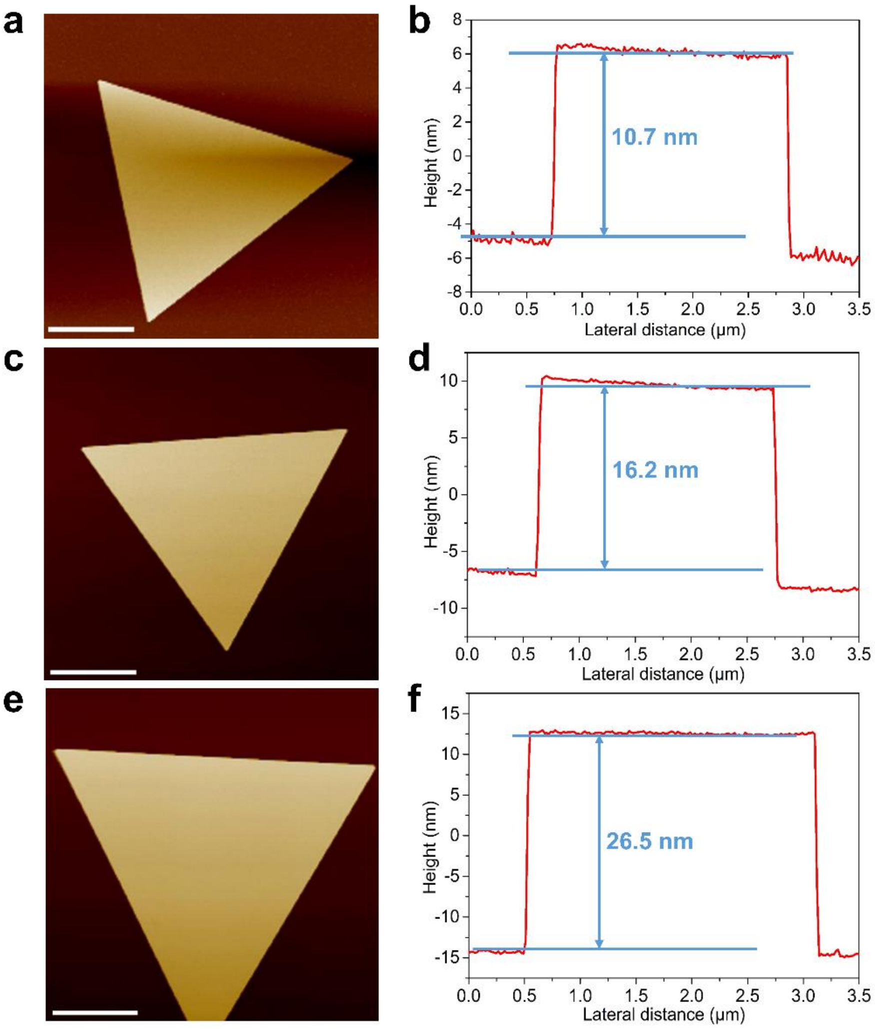

Supplementary Figure 3. AFM images of as-grown Sb_2O_3 flakes with clean and smooth surface. (a,c,e) Representative AFM images of Sb_2O_3 flakes with different thickness. Scale bars are 1 μm. (b,d,f) The corresponding height profiles.

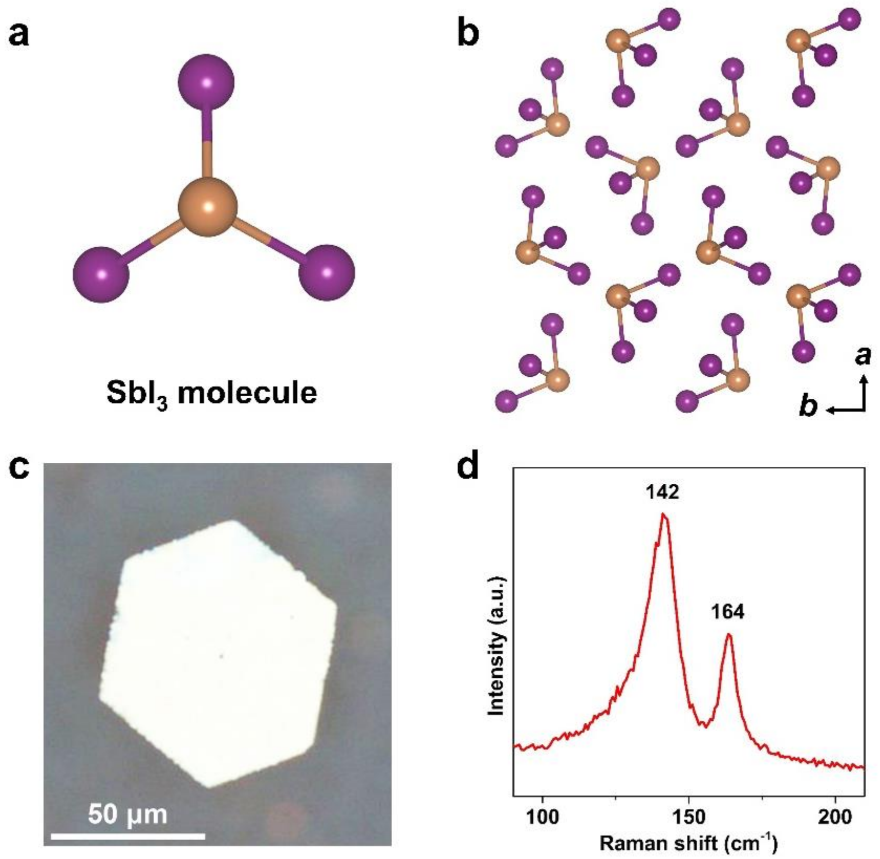

Supplementary Figure 4. Growth of 2D SbI_3 molecular crystals by PAVD. Structural models of SbI_3 molecule (**a**) and SbI_3 molecular crystals (**b**). **c**, Optical image of a typical hexagonal SbI_3 flake deposited on mica. **d**, Raman spectrum of the SbI_3 flakes.

As a typical metal iodide inorganic molecular material, SbI_3 shows a trigonal phase (space group *R*-3). **Supplementary Fig. 4a** shows the molecular structure of SbI_3, which is a typical pyramidal molecule of C_{3v} symmetry. These molecules are combined into SbI_3 molecular crystals by vdW force (**Supplementary Fig. 4b**). Assisted by Se passivator, hexagonal SbI_3 flakes were obtained with the size over 50 µm (**Supplementary Fig. 4c**). As shown in **Supplementary Fig. 4d**, the Raman peaks of the flakes in 142 cm^{-1} and 164 cm^{-1} correspond to A_g and E_g modes of the reported SbI_3 single crystals, respectively, which confirms the synthesis of 2D SbI_3 inorganic molecular crystals.

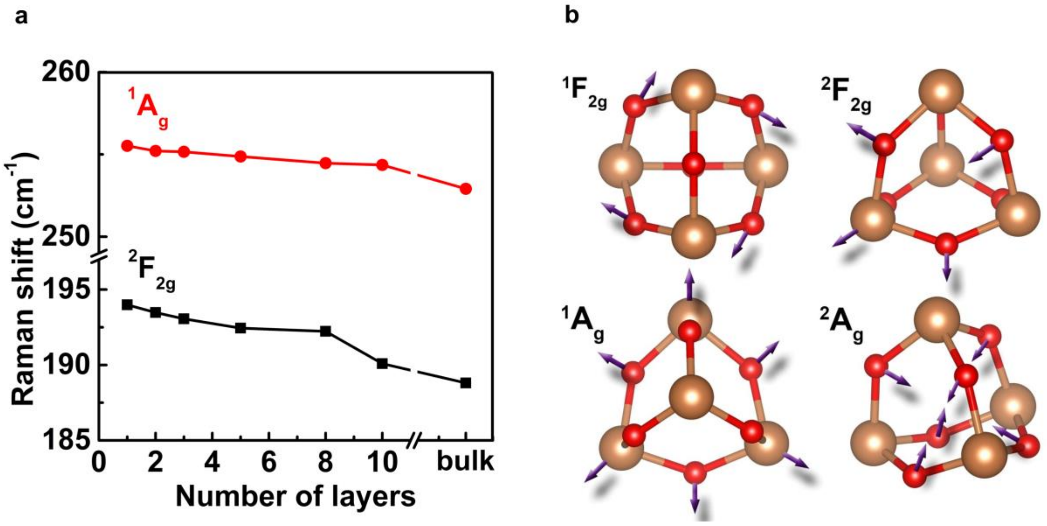

Supplementary Figure 5. Layer-dependent Raman modes and molecular vibration. (a) Peak frequencies of Raman modes $^{1}A_{g}$ and $^{2}F_{2g}$ plotted versus number of layers. (b) Schematic diagrams of atomic displacement (purple arrows) and molecular vibration for modes $^{1}F_{2g}$, $^{2}F_{2g}$, $^{1}A_{g}$, and $^{2}A_{g}$ (brown for Sb atoms, red for O atoms).

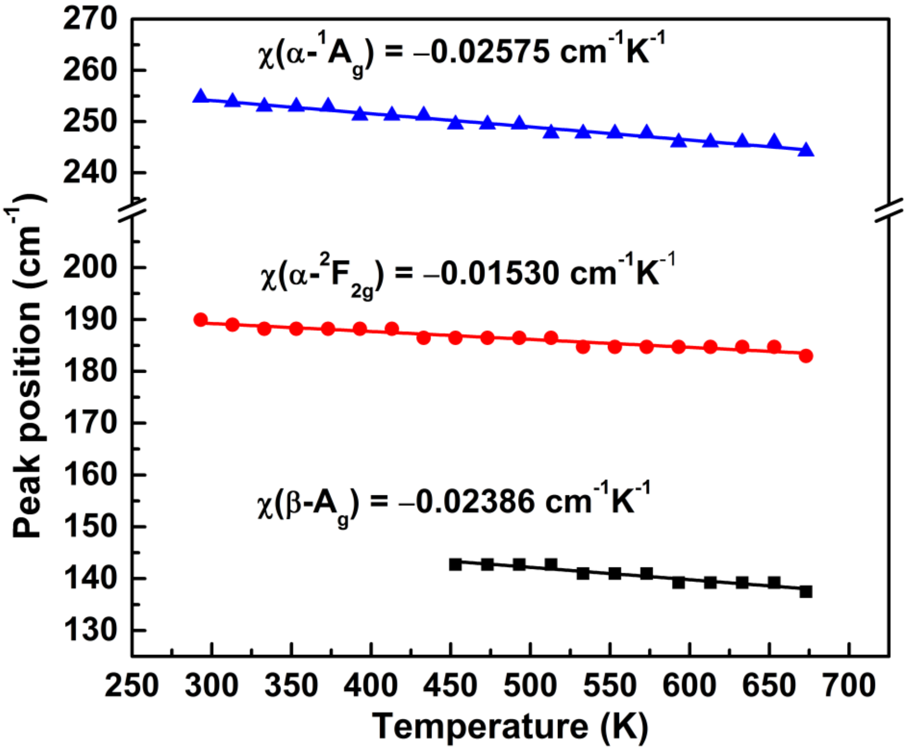

Supplementary Figure 6. Temperature-dependent Raman modes for α and β phases. Temperature dependence of the peak positions of β-A_g, α-$^2F_{2g}$, and α-^1A_g, from bottom to top. The lines are the corresponding fitted lines. The three modes behave nearly linearly with increasing temperature. χ is the first-order temperature coefficient, which also represents the slope of the fitted line. The α-^1A_g has the biggest temperature coefficient, which is the most sensitive mode to the temperature.

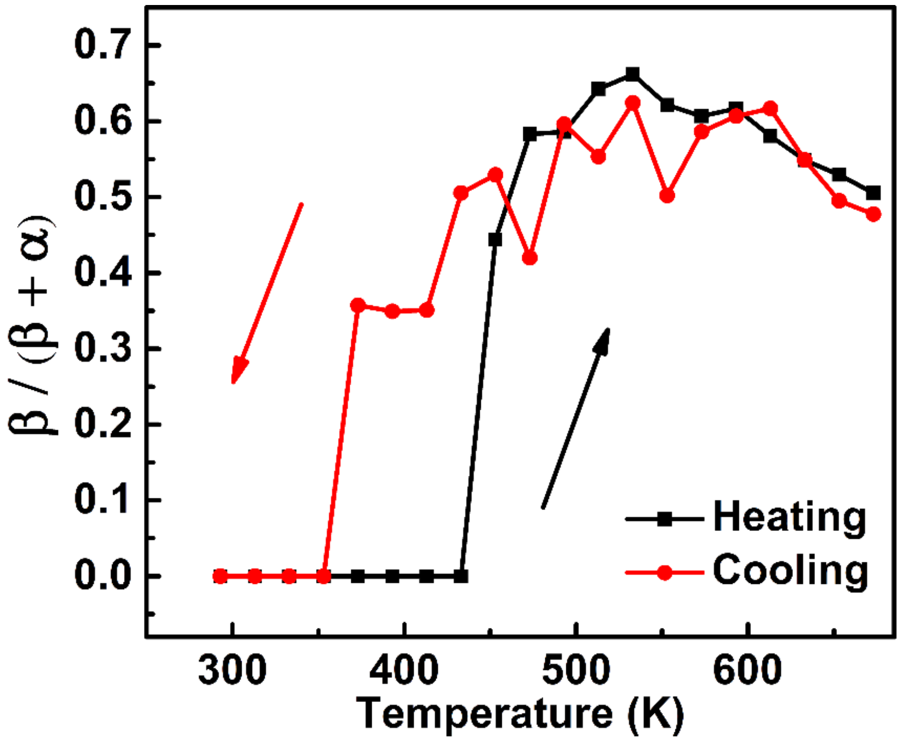

Supplementary Figure 7. Temperature-dependent Raman intensity ratios of two phases showing a thermal hysteresis loop. The ratio R = β(A_g)/[β(A_g)+α(^1A_g)].

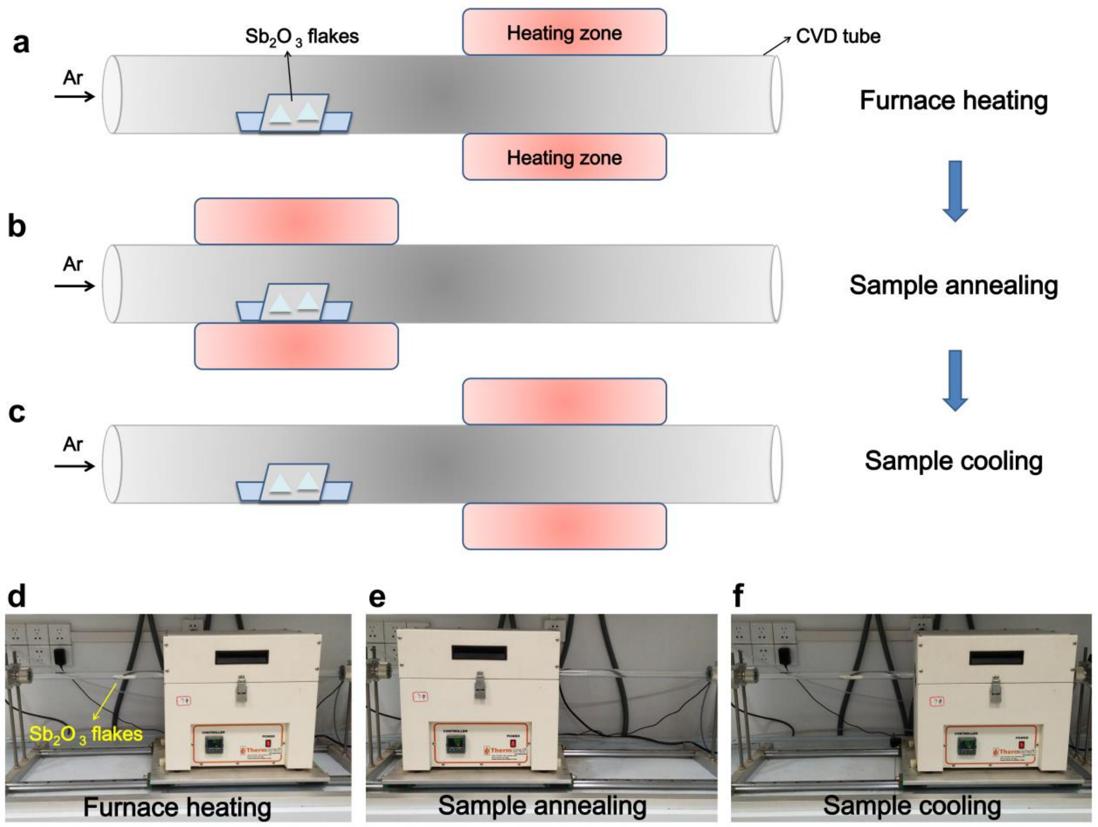

Supplementary Figure 8. Schematic and digital pictures of the rapid annealing process. **(a-c)** Schematic and **(d-f)** the corresponding photos of the rapid annealing process for the phase transition of Sb_2O_3 flakes.

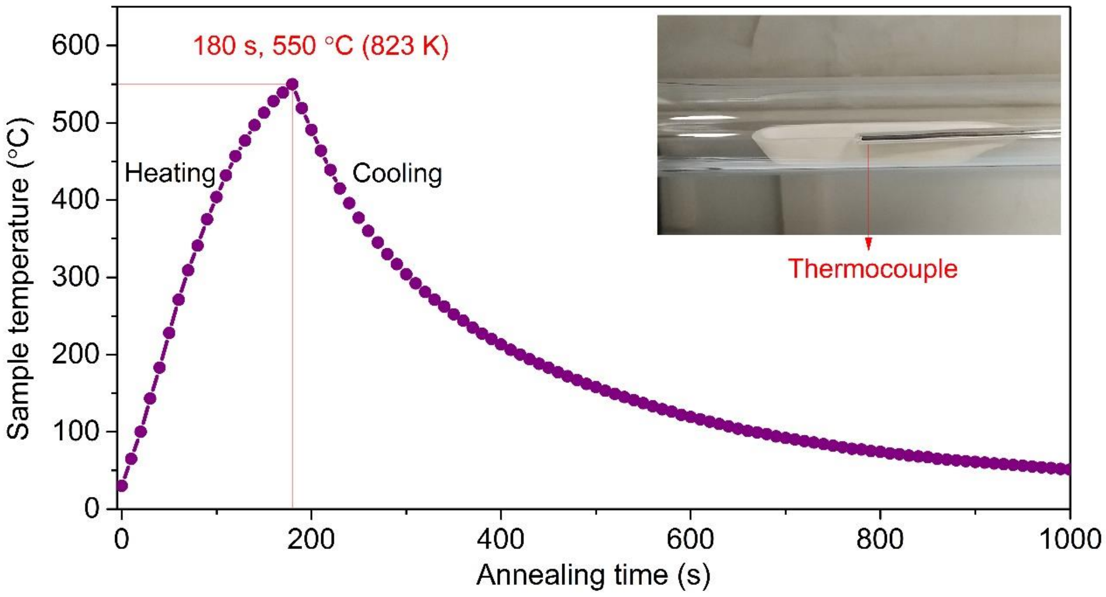

Supplementary Figure 9. Temperature-time curve during the rapid annealing process for the complete phase transition of Sb_2O_3 flakes.

Measurement of ramping rate and cooling rate of annealing

The thermal annealing of Sb_2O_3 flakes were carried out in a single-zone sliding furnace. During annealing, the temperature of the sample was detected in real time by a thermocouple inserted into the furnace near the sample and was monitored by a temperature controller. The temperature-time curves are shown in Supplementary Fig. 9.

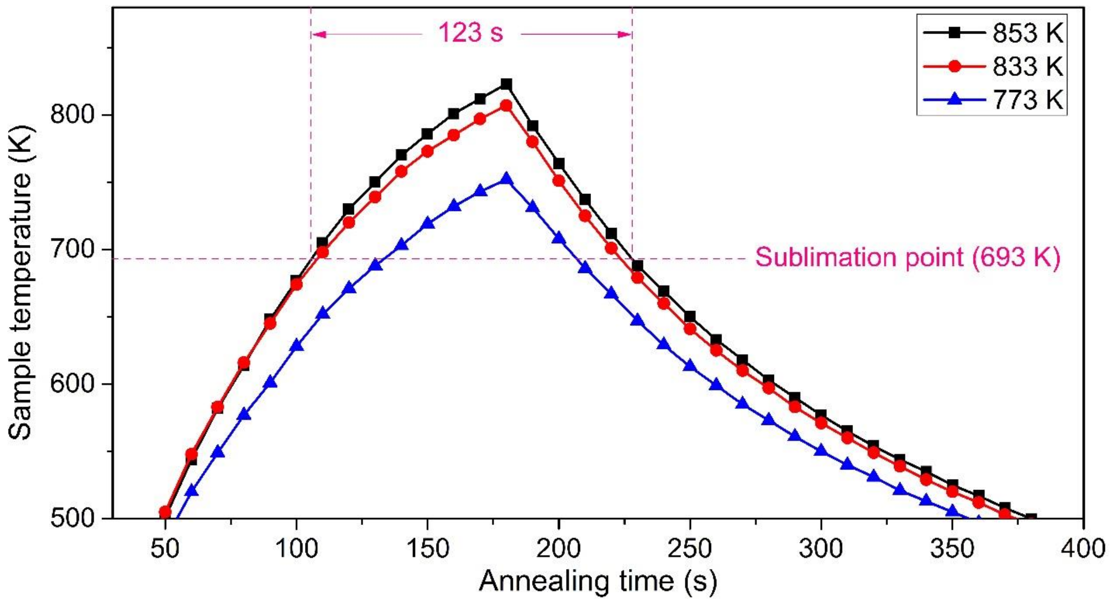

Supplementary Figure 10. Temperature-time curves of the Sb_2O_3 flakes in different annealing temperatures.

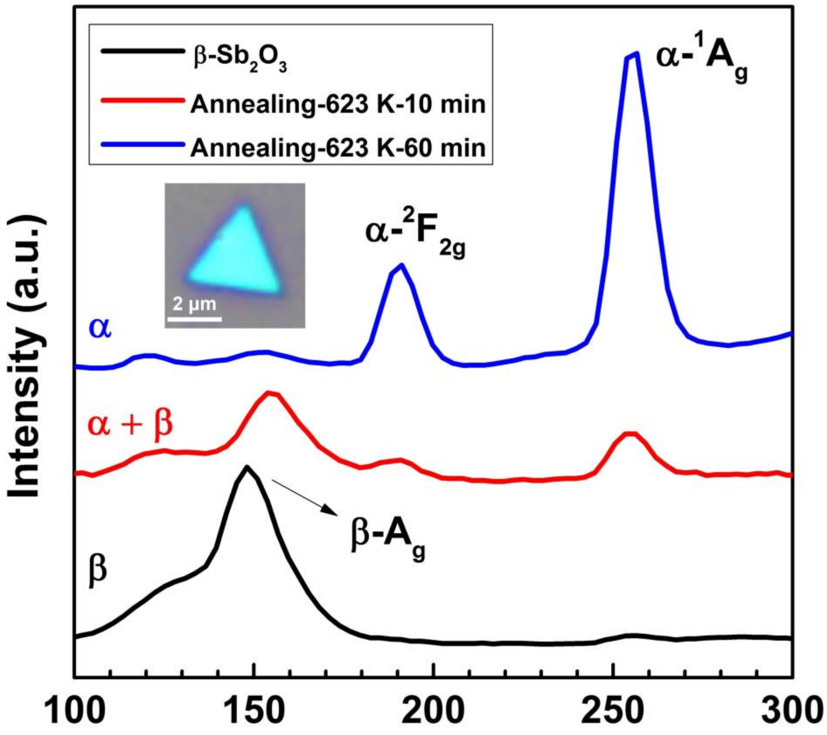

Supplementary Figure 11. Raman spectra of another β-Sb_2O_3 flake before and after annealing for 10 min and 60 min at 623 K. Inset is the optical image of a β-Sb_2O_3 flake after annealing. The evolution of phases with annealing time can be expressed as: β → mixed →α.

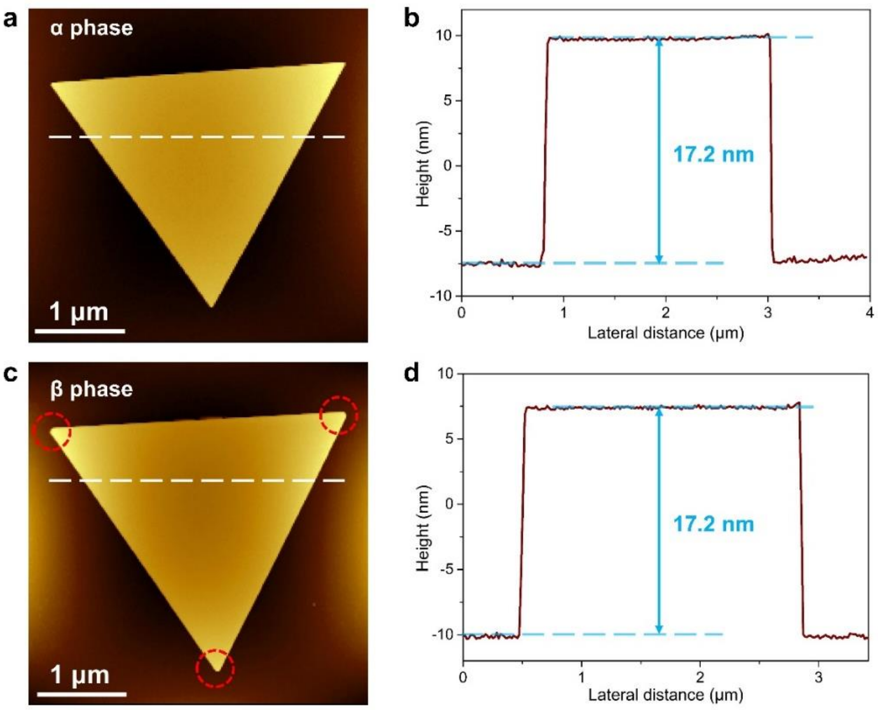

Supplementary Figure 12. AFM images of Sb_2O_3 flake before and after phase transition. (a, c) Representative AFM images of α- and β-phase Sb_2O_3 flake (β-phase: annealing at 823 K for 3 min). (b, d) The corresponding height profiles.

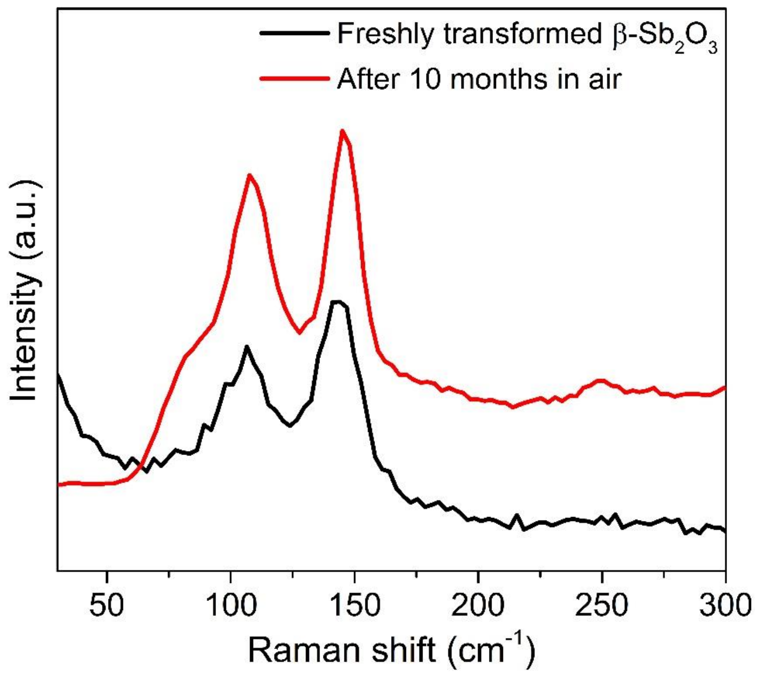

Supplementary Figure 13. Raman spectra of as-transformed Sb_2O_3 flake (test date: 27 May, 2018) and the same sample after 10 months placed in air (test date: 15 April, 2019).

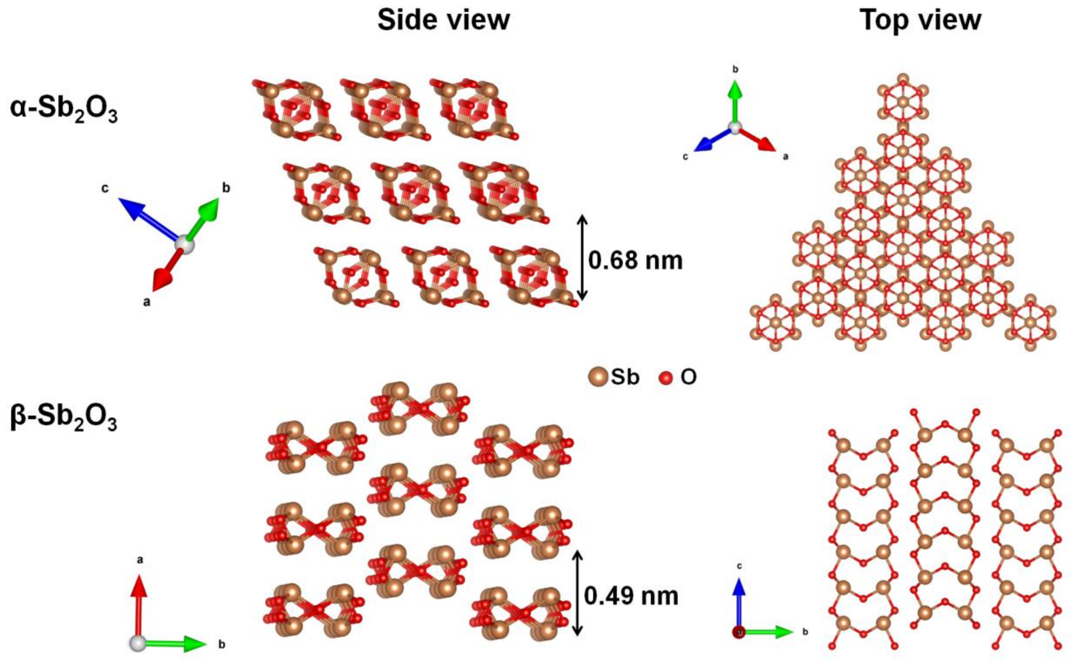

Supplementary Figure 14. The schematic models of α-Sb_2O_3 and β-Sb_2O_3 with side view and top view.

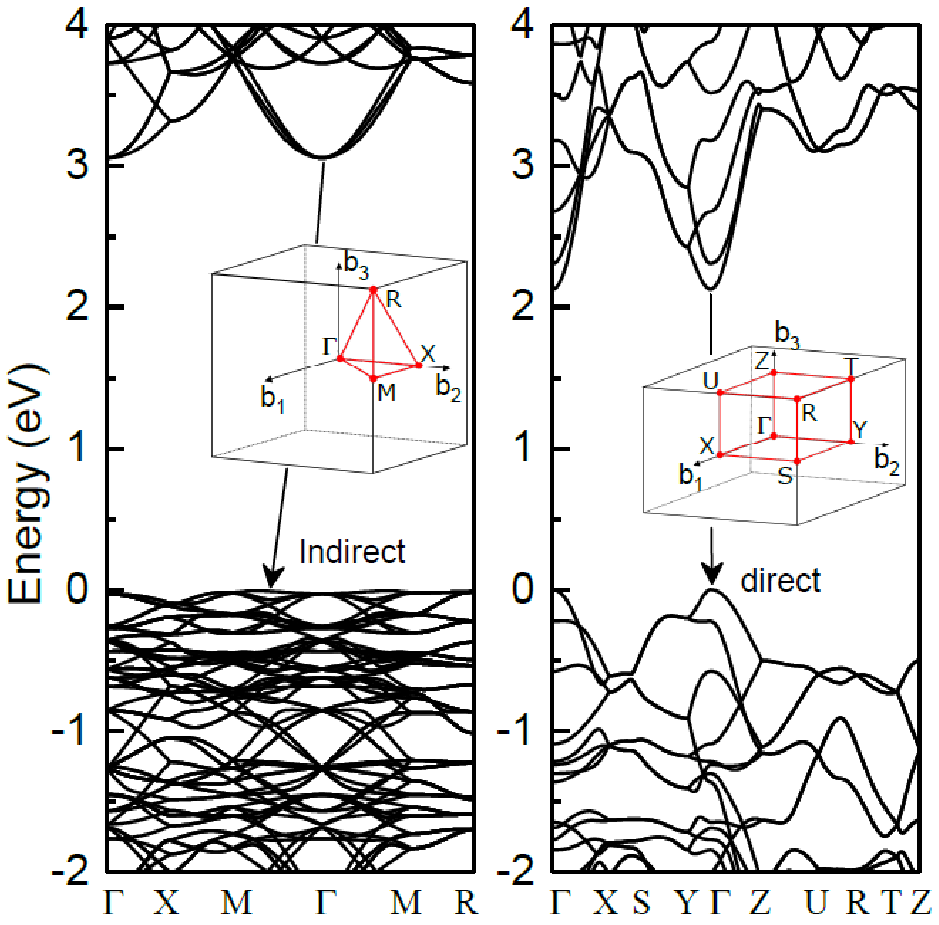

Supplementary Figure 15. Band structures of α-/β- Sb_2O_3 calculated by DFT-PBE. I suggests the distinct transition from indirect bandgap to direct bandgap with the phase change. The significant band dispersion of β-Sb_2O_3 at point indicates the small effective carrier mass thus more favorable for carrier transport in the crystal.

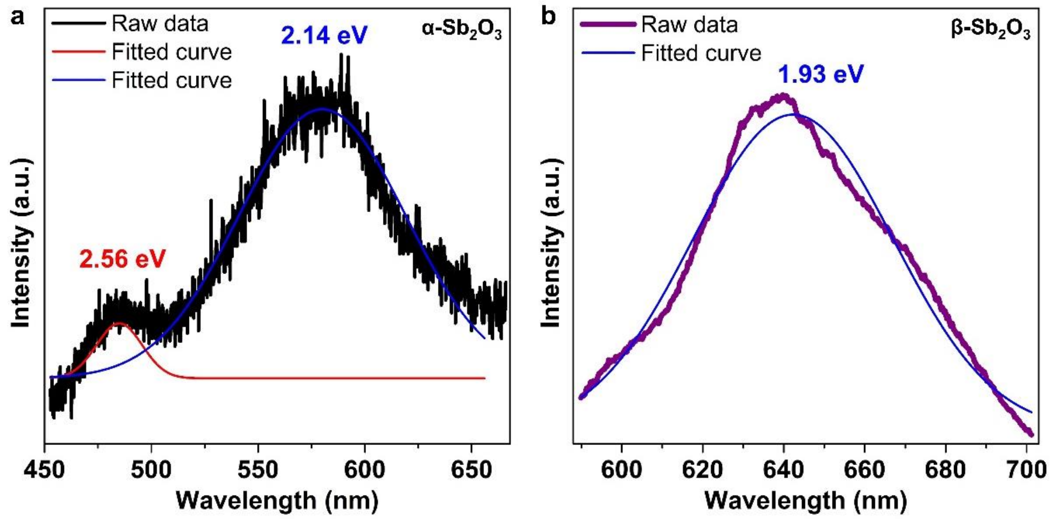

Supplementary Figure 16. Room-temperature PL spectra of α-phase (a) and β-phase (b) Sb_2O_3 flakes.

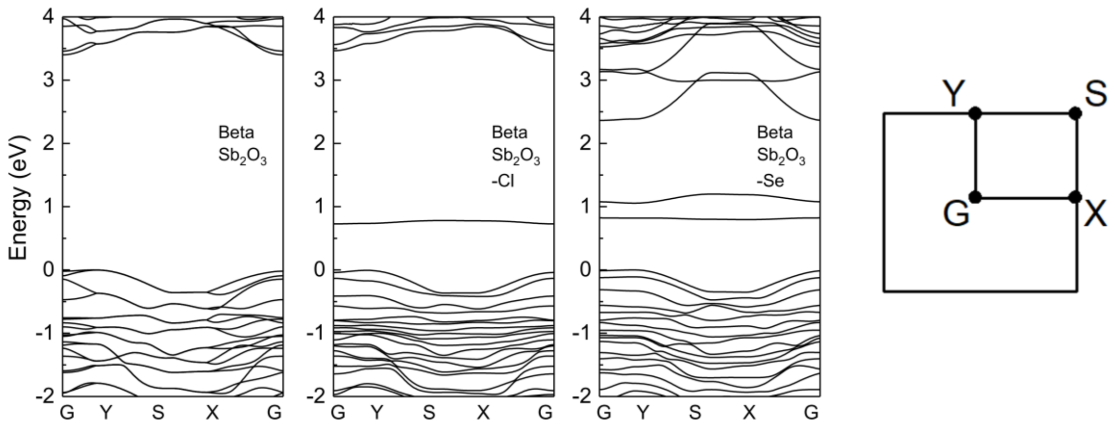

Supplementary Figure 17. The 2D energy bands of beta phase adsorbed by Cl and Se on the surface. The right is Brillouin.

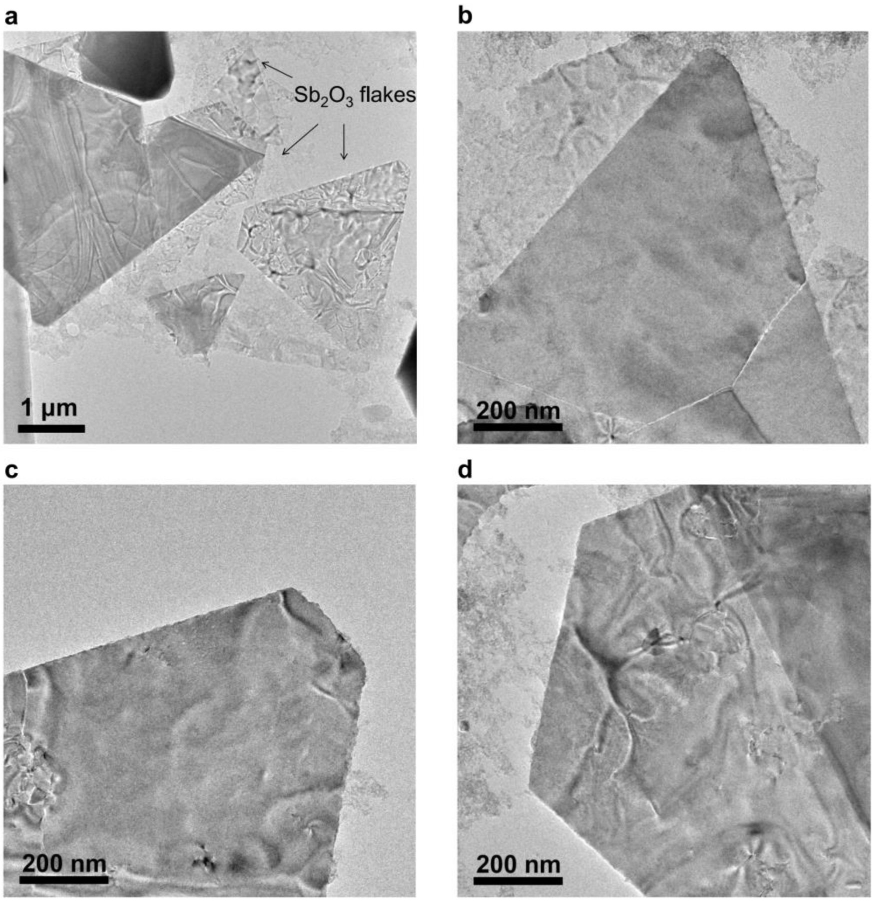

Supplementary Figure 18. TEM images of transferred Sb_2O_3 thin flakes on Si_3N_4 window in a heating chip for in situ TEM. (a) Low magnification image; (b, c, d) Enlarged images.

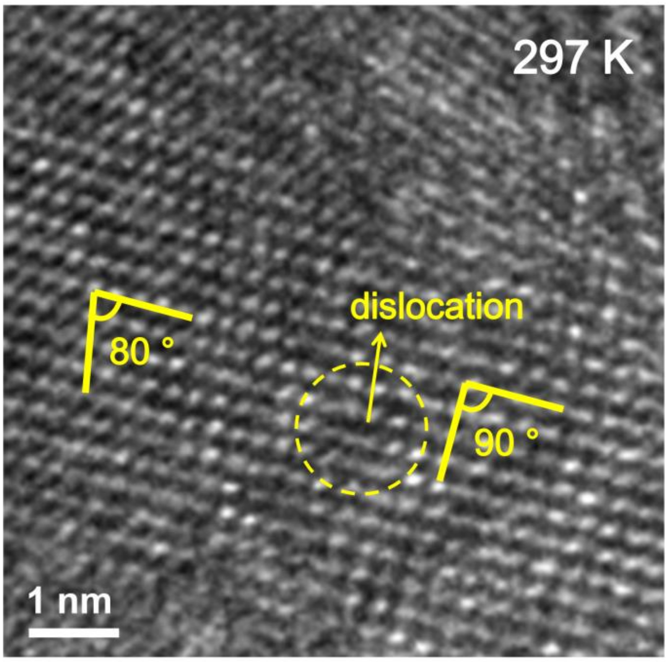

Supplementary Figure 19. TEM image of phase boundary observed in the EBI-induced transition at room temperature.

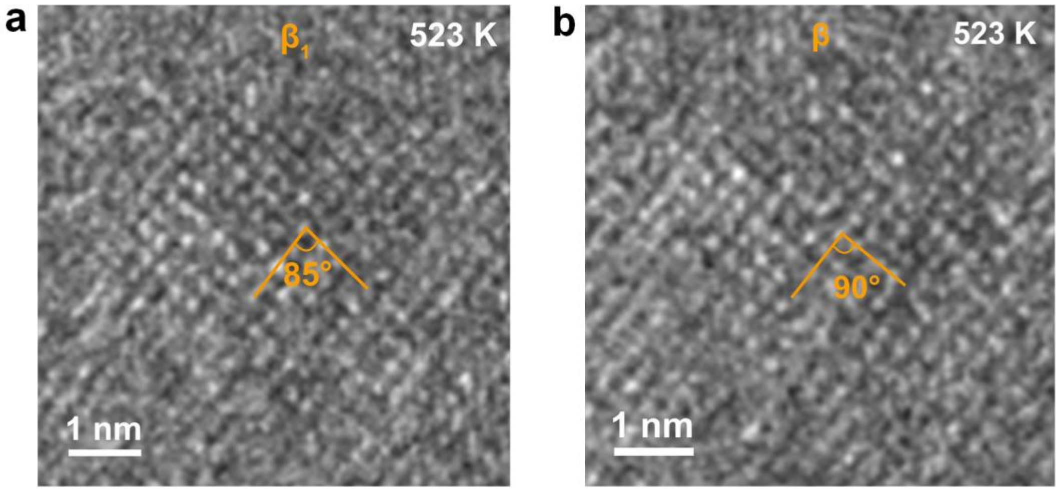

Supplementary Figure 20. HRTEM images of β_1-phase (a) and β-phase (b) in another region of the same Sb_2O_3 flake at 523 K.

Supplementary Table 1. The calibrated annealing temperature and average heating and cooling rates.

The temperature set by the furnace (K)	The temperature of sample after 3 minutes of heating (K)	Average ramping rate (K/min)	Average cooling rate (to 373 K, K/min)
853	823	173.3	56.2
833	807	167	55.4
773	752	146.3	50.5
673	650	117.3	40.5

Supplementary Table 2. The parameters of crystal structures of α- and β-Sb_2O_3 .

	**α**	**β**
**Space group**	*Fd*-3*m* (#227)	*Pccn* (#56)
**Crystal system**	Cubic	Orthorhombic
**Lattice constant**	*a*=11.1519 Å	*a*=4.8996 Å
	b=11.1519 Å	*b*=12.4490 Å
	c=11.1519 Å	*c*=5.4103 Å
	α=90 °	*α*=90 °
	β=90 °	*β*=90 °
	γ=90 °	*γ*=90 °

Supplementary Table 3. Summary of the parameters during the phase transition in Sb_2O_3 flake.

	**α**	**$β_1$**	**β**
**Crystal planes**	(40-4), (4-40)	(040)', (002)'	(040), (002)
**Angle of crystal plane (°)**	60	85	90
**Crystal spacing (nm)**	0.405	0.439	0.540
**Lattice mismatch**	0	0.084	0.333
