# Supplementary material for: Two-dimensional inorganic molecular crystals
Source: Nat Commun. 2019 Oct 17;10:4728. doi: 10.1038/s41467-019-12569-9 (PMC6797790; doi:10.1038/s41467-019-12569-9)
Supplement: Supplementary file 3 — Description of Additional Supplementary Files [file 41467_2019_12569_MOESM3_ESM.pdf]

Description of Additional Supplementary Files

File Name: Supplementary Movie 1
Description: In situ TEM movie of electron-beam irradiation induced $\alpha \rightarrow \beta$ structural transition on the middle of the Sb_2O_3 flake.

File Name: Supplementary Movie 2
Description: In situ TEM movie of electron-beam irradiation induced $\alpha \rightarrow \beta$ structural transition on the edge of the Sb_2O_3 flake.
